# Die Wirkung gemeindenaher, museumsbasierter Aktivitäten für Menschen mit Demenz und ihre primären Betreuungspersonen – ein Umbrella Review

**DOI:** 10.1007/s00391-024-02377-2

**Published:** 2024-12-02

**Authors:** Melanie Kriegseisen-Peruzzi, Mona Dür, Verena C. Tatzer, Piret Paal

**Affiliations:** 1 Doktoratsstudium Nursing & Allied Health Sciences, Institut für Pflegewissenschaft und -praxis, Paracelsus Medizinische Privatuniversität, Strubergasse 21, 5020 Salzburg, Österreich; 2 Duervation, Krems, Österreich; 3 Studiengang Ergotherapie, Fachhochschule Wiener Neustadt, Wiener Neustadt, Österreich; 4 Forschungsinstitut für Palliative Care, Paracelsus Medizinische Privatuniversität, Salzburg, Österreich; 5 Department of Neurobiology, Care Sciences and Society, Div. of Occupational Therapy, Karolinska Institutet, Stockholm, Schweden; 6 BSc-Studiengang Ergotherapie, fhg Fachhochschule Gesundheit GmbH, Innsbruck, Österreich; 7 Abteilung für Ethnologie, Institut für Kulturwissenschaften, Tartu Universität, Tartu, Estland

**Keywords:** Neurokognitive Einschränkung, Psychosozial, Kognitiv, Partizipation, Social prescribing, Neurocognitive impairment, Psychosocial, Cognitive, Participation, Social prescribing

## Abstract

**Hintergrund:**

In den vergangenen Jahren wurde die Wirkung museumsbasierter Angebote für Menschen mit Demenz und ihre primären Betreuungspersonen als gemeindenahe Unterstützungsmöglichkeiten zunehmend beforscht.

**Fragestellung und Ziel:**

Der Umbrella Review führt aktuelle Ergebnisse zur Wirkung museumsbezogener Angebote auf Menschen mit Demenz und ihre primären Betreuungspersonen zusammen und Ieitet Implikationen für die Praxis und die Versorgungsforschung ab.

**Methode und Material:**

Nach den Leitlinien des Joanna Briggs Institute (JBI) wurde ein Umbrella Review auf Basis systematischer Reviews und Metaanalysen erstellt. Die Ergebnisse der systematischen Literaturrecherche in CINAHL Complete, PubMed, Medline Complete, SocINDEX, Psych & Behav Sci sowie die Cochrane Database for Systematic Reviews (April/Mai 2023; Follow-up: Oktober 2023) wurden durch 3 Reviewerinnen kritisch bewertet.

**Ergebnisse:**

Es wurden 5 systematische Reviews und eine Metaanalyse identifiziert. Aufgrund der Heterogenität der primären Studien gibt es derzeit keine konsistenten, statistisch robusten Nachweise zur Wirksamkeit museumsbasierter Angebote. Statistische Tendenzen und qualitative Studien deuten auf eine positive Wirkung auf die Lebensqualität und Stimmung und diverse andere nichtkognitive Parameter hin.

**Diskussion und Fazit:**

Die Heterogenität der bestehenden Forschungsarbeiten und -designs lässt keine statistisch belastbare Aussage zur Wirkung gemeindenaher, museumsbasierter Angebote zu. Die auf eine positive Wirkung hindeutenden Ergebnisse, die auch in diversen randomisierten kontrollierten Einzelstudien berichtet werden, sprechen für Museen als Ressource für Partizipation und für ihre Berücksichtigung im Rahmen des Social Prescribing. Für die weitere Forschung werden angepasste und erweiterte methodische Überlegungen und Ansätze wie Mixed-Methods-Designs empfohlen.

**Zusatzmaterial online:**

Zusätzliche Informationen sind in der Online-Version dieses Artikels (10.1007/s00391-024-02377-2) enthalten.

## Abstract (en)

**Background:**

There has been noticeable research emphasis on the effects of museum-based programs as valuable community support for people with dementia and their primary caregivers.

**Research question and aim:**

This umbrella review outlines the effects of museum-based community programs on health-related aspects of people with dementia and their primary caregivers and aims to synthesise existing evidence of museum-based programs and give directions for practice and implementation research.

**Method and material:**

An umbrella review was undertaken following the guideline of the Joanna Briggs Institute (JBI). Therefore 6 databases (CINAHL Complete, PubMed, Medline Complete, SocINDEX, Psych & Behav Sci and the Cochrane Database for Systematic Reviews) were searched for systematic reviews and meta-analyses in April/May 2023 and as a follow-up in October 2023. The results were critically appraised by three reviewers.

**Results:**

A total of five systematic reviews and one meta-analysis were identified. Due to the heterogeneity of the primary studies no consistent statistically robust proof of effects of museum-based community programs currently exists. Qualitative studies support the effects towards improvement of quality of life, mood and other noncognitive parameters.

**Discussion and conclusion:**

While the heterogeneity of study designs prevents a critical evaluation of the effects and therefore a reliable comprehensible analysis, the results from different studies, including randomized controlled trials, support museum-based programs as a valuable resource for participation in day to day care and social prescribing. For future research adapted and extended methodological considerations and approaches, such as mixed methods designs are recommended.

## Hintergrund

Demenzen gehören zu den häufigsten Ursachen für Einschränkungen der biopsychosozialen Gesundheit, des Wohlbefindens und der Partizipation sowohl der Betroffenen als auch ihrer primären Betreuungspersonen [21]. Die demografisch bedingte Zunahme von Betroffenenzahlen in den kommenden Jahren verlangt somit besonderes Augenmerk auf die Bedürfnisse dieser Personengruppen [11, 21]. Die Teilhabe am öffentlichen Leben, insbesondere das Aufrechterhalten gewohnter Freizeitaktivitäten in bedeutungsvollen räumlichen Kontexten [10], ist bereits früh im Krankheitsverlauf eingeschränkt [17]. Die Weltgesundheitsorganisation [21] ebenso wie die nationale Demenzstrategie Österreich fordern, den Lebenskontext Betroffener und ihrer Angehörigen zu verbessern und die vielschichtigen Auswirkungen der Erkrankung zu verringern, indem Möglichkeiten geschaffen werden, die das Leben mit Demenz in der Gemeinschaft ermöglichen und die Betreuungslast reduzieren [11, 21].

Im deutschsprachigen Raum wird derzeit das Social Prescribing diskutiert, das als innovativer Versorgungsansatz systematisch die psychosozialen und emotionalen Anliegen von Menschen mit unterschiedlichen gesundheitlichen Themenstellungen adressiert und Angebote der Primärversorgung mit Angeboten von regionalen kommunalen und sozialen Stakeholdern vernetzt. Museen sind dabei als Partnerbetriebe für die gemeindenahe Versorgung von Menschen mit Demenz (MmD) vorgesehen [18]. Aufgrund der Forschungsergebnisse der vergangenen Jahre kann davon ausgegangen werden, dass Museumsbesuche positive Auswirkungen auf Aspekte der biopsychosozialen Gesundheit und des Wohlbefindens von Betreuungsdyaden, bestehend aus MmD und ihren primären Betreuungspersonen, haben [4, 6, 13]. Vor diesem Hintergrund geht der Umbrella Review folgender Forschungsfrage nach: Welche Wirkung haben gemeindenahe, museumsbasierte Angebote auf gesundheitsbezogene Aspekte von Menschen mit Demenz und ihre primären Betreuungspersonen?

Ziel ist es, Erkenntnisse, die in Form von systematischen Reviews und Metaanalysen vorliegen, zusammenzuführen und die Wirkung museumsbasierter Angebote auf die biopsychosoziale Gesundheit und das Wohlbefinden von MmD und ihren Angehörigen aufzuzeigen. Zudem werden aus der Literatur Impulse für die zukünftige Praxis- und Versorgungsforschung und Gerontologie abgeleitet [2].

## Methode

Die Grundlage für den Umbrella Review bilden die Empfehlungen des Joanna Briggs Institute Manual for Evidence Synthesis (JBI-MES) [2]. Die Richtlinien von Fusar-Poli und Radua [9] wurden als Orientierungshilfe herangezogen. Nach einer orientierenden Literaturrecherche waren Ergebnisse in Form von quantitativen und qualitativen Reviews zu erwarten. Entsprechend dem JBI-MES [2] wurde daher eine PICO- (Population, Intervention, Comparison, Outcome) als auch eine PICo- (Population, Phenomena of Interest, Context) Suchstrategie verfolgt.

Als Datenbanken für die systematische Literaturrecherche wurden CINAHL Compl., PubMed, Medline Compl., SocINDEX, Psych & Behav Sci sowie die Cochrane Database for Systematic Reviews ausgewählt. Nach abgeschlossener elektronischer Suche wurden die Quellenangaben der ausgewählten Übersichtsarbeiten sowie verfügbare „graue Literatur“ auf den Homepages unterschiedlicher Stakeholder manuell nach weiteren Ergebnissen durchsucht. Die Suche erfolgte mit englischsprachigen Schlagwörtern unter Verwendung des Extender „all languages“, was durch die Sprachkenntnisse der Autorinnen auch Studien in anderen Sprachen zugänglich gemacht hätte.

Als Suchbegriffe (ggf. trunkiert) kamen „museum“, „art gallery“, „cultural“, „non-pharmacological“, „psychosocial“, „dementia“, „cognitive impairment“, „memory loss“, „caregiver“ sowie „systematic review“, verbunden durch die Bool-Operatoren AND und OR zum Einsatz. Zusätzliche Filter waren „Abstract und Titel“, „Systematic Review“, „Peer-reviewed“ und „Verwandte Begriffe“ [2].

Die systematische Literaturrecherche wurde von der Erstautorin im April und im Mai 2023 durchgeführt, im Oktober erfolgte eine systematische Follow-up-Recherche anhand der Suchprotokolle der ersten Recherche.

Die kritische Bewertung der Volltexte wurde von 3 Autorinnen (M. K.P., M.D., V. C.T.) anhand der „JBI Checklist for Systematic Reviews and Research Syntheses“ [1] unabhängig voneinander durchgeführt. Divergenzen wurden im Autorinnenteam bis zur Einstimmigkeit diskutiert.

Die Datenextraktion erfolgte durch die Erstautorin mithilfe einer an das „JBI Data Extraction Form for Review for Systematic Reviews and Research Syntheses“ [14] angelehnten Tabelle. Neben Autor:innenschaft, Publikationsjahr, Titel, Methode und Anzahl der inkludierten Studien enthält diese Informationen zu Forschungsfrage und -ziel, Kontext, Population, Interventionen/Maßnahmen sowie zentrale Ergebnisse.

Eingeschlossen wurden Übersichtsarbeiten, die gemeindenahe Angebote in bzw. in Zusammenarbeit mit Museen für zu Hause lebende Menschen mit Demenz und ihre primären Betreuungspersonen mit Fokus auf deren psychosoziale Aspekte, Kognition, Lebensqualität und Wohlbefinden untersuchten.

## Ergebnisse

Abb. 1.

**Abb. 1:**
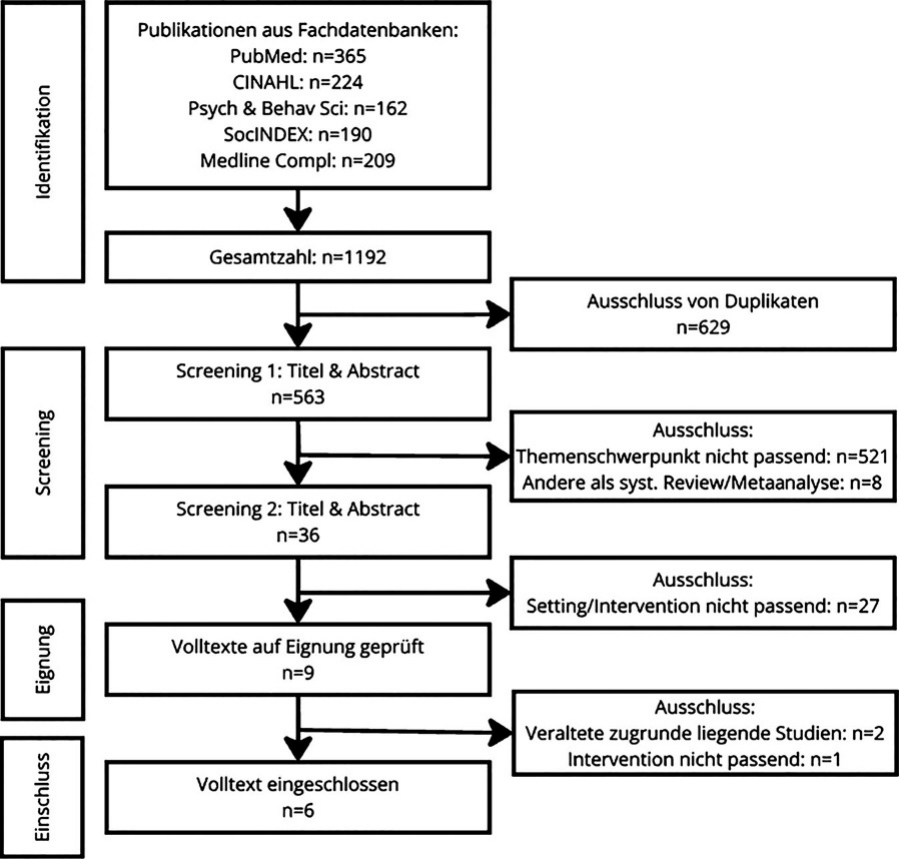
Flussdiagramm: systematische Literaturrecherche, Oktober 2023

Die Ergebnisse des Umbrella Review wurden aus 5 systematischen Reviews und einer Metaanalyse (publiziert zwischen 2016 und 2023) generiert. Bei 4 der 5 systematischen Reviews [3, 8, 16, 22] reicht das Spektrum der Interventionen von kulturbezogenen Angeboten wie Tanz und Literatur bis hin zu museumsbasierten Angeboten. Die unterschiedlichen Interventionen, so auch museumsbasierte Angebote, wurden in diesen Übersichtsarbeiten jeweils separat auf ihre Wirkung untersucht. Den alleinigen Fokus auf museumsbasierte Angebote bieten Zeilig et al. [23], der Fokus auf Angehörige und Personen mit Demenz findet sich bei Delfa-Lobato et al. [8] und Bourne et al. [3]. Die inkludierten systematischen Reviews berücksichtigen quantitative, qualitative und Mixed-Methods-Design-Studien. Die Metaanalyse [7] basiert auf randomisierten und quasirandomisierten Studien und betrachtet die Auswirkungen von kulturbasierten Angeboten auf Menschen mit kognitiven Einschränkungen. Sie beinhaltet neben einer den gesamten Kultursektor umspannenden Metaanalyse auch partielle Metaanalysen, die Ergebnisse zu Interventionsformen, Evaluierungsaspekten, Formen der kognitiven Einschränkungen (CI) und unterschiedlichen Moderatorvariablen liefern. Museumsbesuche fallen hierbei unter den Sammelbegriff „Visual Arts“.

**Abb. 2:**
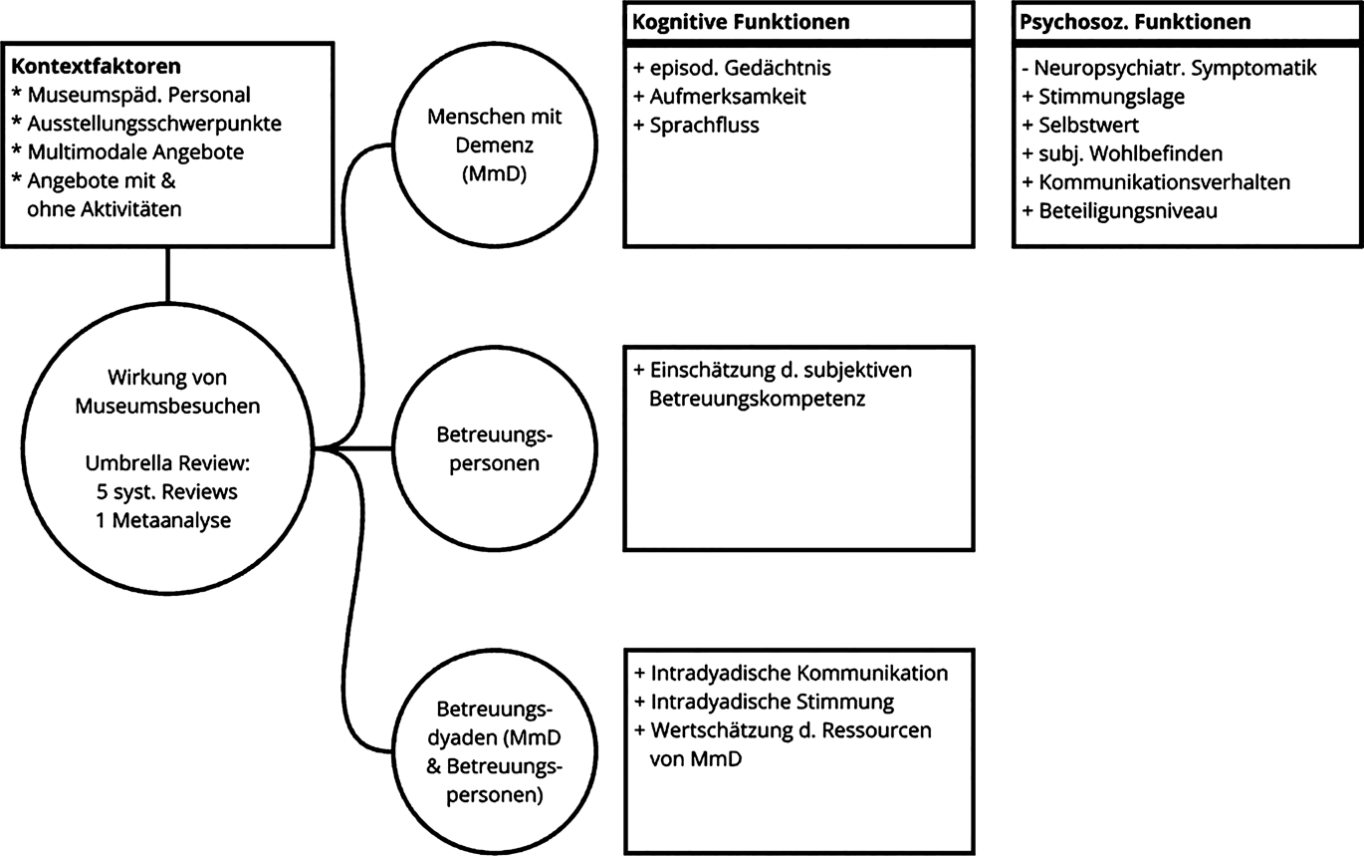
Darstellung der Ergebnisse

Die Effekte wurden in Bezug auf die Betroffenen, deren Angehörige und auf die subjektiv erlebte Beziehung innerhalb der Betreuungsdyade beschrieben (Abb. 2). Zusätzlich wird der Kontext der identifizierten Interventionen dargestellt. Details zu den Ein- und Ausschlusskriterien, zur kritischen Bewertung und zur Datenextraktion finden sich im Zusatzmaterial online.

### Menschen mit Demenz

#### Kognitive Funktionen

Die systematischen Reviews beschreiben positive Veränderungen in den Bereichen Gedächtnis, Aufmerksamkeit und Sprachfluss [3, 8, 16, 22, 23] sowie zusätzlich Verbesserungen der „allgemeinen Kognition“ [8], die nicht weiter spezifiziert wird. Alle Arbeiten berichten, dass bisher nur in wenigen der analysierten Einzelstudien signifikante Effekte gemessen wurden, die in Follow-up-Designs nicht feststellbar waren. Die Metaanalyse [7] ergab über die gesamten kulturbasierten Interventionen schwache signifikante Effekte (ω = 0,10, mit KI [0,02, 0,18], mit *p* = 0,02); der Heterogenitätstest bestätigte die hohe Heterogenität der unterschiedlichen Studien (Q = 1622,56, *p* < 0,01, I2 = 91,13 %).

#### Psychosoziale Aspekte

Museumsbasierte Angebote wirken sich laut der Ergebnisse der systematischen Reviews positiv auf das Wohlbefinden der Menschen mit Demenz aus [3, 8, 16, 22, 23]. So werden eine allgemeine Stimmungsaufhellung, stabilere Stimmungslagen sowie erhöhter Selbstwert beschrieben [16]. Anhand von Selbst- und Fremdbeurteilung wurde eine Steigerung von subjektivem Wohlbefinden nachgewiesen [16, 23]. Zudem finden sich qualitative und quantitative Studien, die mit der Verbesserung der Lebensqualität [8], der Reduktion neuropsychiatrischer Symptome wie Apathie, Depression und Angst [3, 8], psychosozialen Aspekten und Aspekten von Lebensqualität und Wohlbefinden assoziiert sind [3, 8, 22].

Zur Lebensqualität fanden sich keine signifikanten Effekte (ω = 0,25, KI [0,67, 0,17], mit *p* = 0,25) bei ebenfalls hoher Heterogenität (Q = 190,01, *p* < 0,01, I2 = 96,48 %).

Die Analyse der Moderatorvariablen ergab statistisch signifikante Zusammenhänge zwischen den KI des höheren Alters und der Effektstärke (b = 0,221, mit *p* = 0,002), die darauf hinweisen, dass bei jüngeren Personen dieser Gruppe höhere Effektstärken durch kulturbasierte Angebote zu erwarten sind.

Mehrere Reviews fassen Angaben zu spontaner bzw. vermehrter Kommunikation von Menschen mit Demenz zusammen [3, 8, 22]. Außenbeobachtungen durch die Betreuungspersonen beschreiben zudem positive Veränderungen der Kommunikation und des Beteiligungsniveaus [16].

### Primäre Betreuungspersonen

Aussagen über eine verbesserte Einschätzung der eigenen Betreuungskompetenz bei Angehörigen finden sich in einer systematischen Übersichtsarbeit [16].

### Betreuungsdyade

Vier der systematischen Reviews [3, 8, 16, 23] beschreiben verbesserte Kommunikation und Stimmung zwischen Menschen mit Demenz und ihren Angehörigen und führen dies auf die miteinander verbrachte Zeit im Rahmen von geschätzten Aktivitäten zurück [8, 16]. Qualitative Studien liefern Hinweise dafür, dass museumsbasierte Aktivitäten potenziell empfunden werden als „Normalität“, die es betreuenden Angehörigen ermöglicht, die Fähigkeiten von Menschen mit Demenz expliziter wahrzunehmen [3].

### Kontextfaktoren

Alle eingeschlossenen Studien fanden in Museen und weitgehend unter der Leitung museumspädagogisch geschulter Personen statt, in Einzelfällen in gemeindenahen Institutionen in Zusammenarbeit mit örtlichen Museen [7]. Museumsbasierte Aktivitäten wurden mit oder ohne bildnerische Aktivitäten, für unterschiedliche Kunstrichtungen bzw. in unterschiedlichen Modalitäten angeboten. Mehrere Reviews bestätigen die positive Wirkung von Museumsbesuchen bei Menschen mit Demenz unabhängig von Angebotsform und Modalität [3, 8, 16]. Qualitative Studien in Bezug auf die Betreuungsdyaden und die Betreuungspersonen unterstützen die Aussagen über die Wirkung von museumsbasierten Angeboten zusätzlich [16]. Als besonders unterstützend werden in den Reviews Aktivitäten in Kombination mit Kommunikation angeführt. Dem Austausch in der Peer-Group und dem gemeinsamen Ausführen der Aktivitäten innerhalb der Betreuungsdyade, aber auch innerhalb von homogenen Gruppen wird positiver Einfluss auf Wohlbefinden und Kommunikation zugesprochen [3, 8].

### Qualität und Bias

Für die Datensynthese liegt aktuelle Literatur vor, die sich nicht nur hinsichtlich des Fokus, sondern zusätzlich hinsichtlich der Autorenschaft überschneidet. So findet sich in 3 Übersichtsarbeiten jeweils ein gemeinsamer Co-Autor [3], die 3 Autorinnen eines Reviews [8] sind Teil des Autorenteams der einbezogenen Metaanalyse [7]. Bei der Durchsicht der 5 eingeschlossenen systematischen Übersichtsarbeiten fällt auf, dass diese sich hinsichtlich ihrer Datengrundlage teilweise überschneiden; für die Metaanalyse [7] gilt dies nicht.

Es zeigt sich, dass die Zahl aussagekräftiger Studien wenige Jahre nach Eröffnung dieses Forschungsbereiches aktuell noch begrenzt und die Heterogenität der einzelnen Forschungsprojekte hinsichtlich Anzahl und Merkmale der Teilnehmenden, Methodik, Forschungsdesign sowie Gestaltung der beforschten museumsbasierten Programme groß ist. Laut einstimmiger Kritik aller 5 Reviews finden sich bei weitgehend guter Qualität der in ihre Arbeiten eingeschlossenen Studien nur wenige, die in ihren Messungen und Designs vergleichbar wären [3, 8, 16, 22, 23]. Die Heterogenität der verfügbaren Datenlage bestätigt auch die Metaanalyse [7]. Alle eingeschlossenen Arbeiten basieren fast ausschließlich auf Studien mit Menschen mit leichter bis moderater Demenz, über die Wirksamkeit von museumsbasierten bzw. kunst- und kulturbasierten Angeboten für Menschen mit fortgeschrittenen neurokognitiven Einschränkungen sowie für deren primäre Betreuungspersonen liegen damit aktuell keine Studienergebnisse in Form von systematischen Reviews vor.

## Diskussion

### Diskussion der Ergebnisse

Demenzen unterschiedlicher Ätiologie zeichnen sich durch progrediente Einschränkungen neurokognitiver Funktionen aus. Weder die inkludierten systematischen Reviews [3, 8, 16, 19, 22] noch die Metaanalyse [7] liefern konsistente, statistisch robuste Ergebnisse für die positive, verbessernde Wirkung museumsbasierter Angebote auf die neurokognitiven Funktionen von Menschen mit leichter oder moderater Demenz. Übereinstimmend mit den Aussagen aller Autorenteams zu methodischer und inhaltlicher Heterogenität der ihren Übersichtsarbeiten zugrunde liegenden Studien lassen sich diese Ergebnisse vor unterschiedlichen Hintergründen diskutieren:

Die beforschten Angebote für MmD und ihre primären Betreuungspersonen sind, soweit eruierbar, Gruppenprogramme, die von Forschungsgruppen für bzw. zusammen mit Kunst- und Kulturinstitutionen entworfen und geplant wurden und sind daher in Inhalt, Aufbau und Schwerpunkt nicht vergleichbar. Gleiches gilt für die eingesetzten Mess- und Evaluierungsinstrumente. Das macht eine Vergleichbarkeit der Ergebnisse und infolge eine Übertragbarkeit auf die Population der MmD auf Basis der aktuellen Studienlage weitgehend unmöglich. Die schwach signifikanten Effekte der Metaanalyse hinsichtlich kognitiver Funktionen beziehen sich nicht ausschließlich auf den inhaltlichen Angebotsschwerpunkt „Museum und bildende Kunst“ und können somit nur als „positiver Richtwert“, nicht aber als belastbare Aussage zur verbessernden Wirkung von museumsbasierten Angeboten interpretiert werden.

Durch die bekannte Progredienz und Individualität der neurokognitiven Symptomatik einer Demenz stellt sich die Frage, in welchem Ausmaß von einer verbessernden Wirkung von museumsbasierten Angeboten ausgegangen werden bzw. wie diese mit den derzeit zur Verfügung stehenden (Gold‑)Standards gemessen werden kann [20].

Ähnlich gelagert sind die Ergebnisse zur Wirkung museumsbasierter Angebote auf psychosoziale Aspekte. Die beschriebenen Verbesserungen neuropsychiatrischer Symptome, ebenso wie verbesserte kommunikative Fähigkeiten, höherer Beteiligungsgrad, Selbstwert und Wohlbefinden sind nur begrenzt statistisch belastbar; auch hier liefert die Metaanalyse statistisch signifikante Überblickswerte ausschließlich über das gesamte Spektrum der kulturbasierten Angebote.

Vergleichbar mit den Studien zur Verbesserung kognitiver Funktionen kommt auch bei der Messung psychosozialer und kommunikativer Fähigkeiten sowie für Wohlbefinden und Lebensqualität eine erhebliche Bandbreite von Instrumenten zum Einsatz. Konzepte wie Lebensqualität und Wohlbefinden werden unterschiedlich operationalisiert, was die Vergleichbarkeit der Ergebnisse zusätzlich erschwert.

Die positiven Ergebnisse für die intradyadische Kommunikation und für primäre Betreuungspersonen stammen großteils aus qualitativen Studien und weisen darauf hin, dass Aktivitäten, die von beiden Teilen der Betreuungsdyade geschätzt werden, als positiv empfunden werden. Die Ergebnisse zu möglichen wirksamen Kontextfaktoren lassen schließen, dass extern angeleitete (Gruppen‑)Aktivitäten mit hohem Kommunikationsanteil in einem neutralen, positiv konnotierten Kontext als Ressource im Betreuungsalltag gelten können. Diese, wenngleich statistisch nichttragfähigen, Aussagen unterstützen die Forderung der WHO nach unterstützenden Umwelten für MmD und ihre primären Betreuungspersonen [21].

Um museumsbasierte Aktivitäten für MmD und ihre primären Betreuungspersonen im Rahmen von Social Prescribing und Public-Health-Programmen zugänglich zu machen, ist es grundlegend, ihre Wirkung besser zu belegen.

Ausgehend von museumsbasierten Angeboten als „komplexe Intervention“ [5] erscheint eine Verlagerung der Forschungsmethodik auf Mixed-Methods-Designs mit einer Kombination von Pre-post- und Follow-up-Tests mit qualitativen Daten sinnvoll. Aufgrund der Heterogenität der Studiendesigns, insbesondere von Follow-up-Studien, können von bisherigen Studien keine validen Aussagen zur Nachhaltigkeit von museumsbasierten Angeboten abgeleitet werden [3, 16, 23]. Voraussetzung für die Vergleichbarkeit von zukünftigen Forschungsvorhaben sind veränderungssensible, theoriebasierte Messinstrumente und -methoden, die den Ansprüchen kunstbezogener Interventionsangebote gerecht werden und mit bereits bestehenden „Goldstandards“ der neurokognitiven und psychosozialen Diagnostik kompatibel sind [3, 22]. Dergleichen sind derzeit noch nicht verfügbar und müssen mit Blick auf eine entsprechende Operationalisierung von kognitiven und psychosozialen Parametern und vergleichbarere Studiendesigns erst entwickelt werden [3, 8].

Grundsätzlich muss davon ausgegangen werden, dass interessenbezogene und räumlich vorbestimmte Angebote in Museen nur für einen Teil der Zielgruppe „Menschen mit Demenz und ihre primären Betreuungspersonen“ relevant sind. Mit Blick auf die Ergebnisse der Metaanalyse [7] hinsichtlich der potenziell besseren Wirksamkeit von kulturbezogenen Angeboten bei „jüngeren Alten“ sollten auch demografische Daten wie Bildungshintergrund, Wohnlage und räumliche Anbindung sowie Alter erhoben und in die Ergebnisauswertung einbezogen werden [3, 16, 22, 23]. Mit Blick auf kostengünstige, gemeindenahe Unterstützungsangebote ist auch das Einbeziehen von Betreuungsdyaden mit Menschen mit fortgeschrittener Demenz notwendig [22].

Damit ergeben sich für die Planung und Implementierung von museumsbasierten Angeboten zusätzliche Implikationen. Die in die systematischen Reviews inkludierten Studien beforschten Angebote, die von Forschenden und/oder Museumsbetrieben für die Zielgruppe entwickelt wurden. Damit muss von einer Limitierung der Zugänglichkeit auf Personen, die sich speziell für diese Programme interessierten, ausgegangen werden. Als notwendig für die zukünftige Entwicklung von zielgruppenorientierten, demenzgerechten museumsbasierten Programmen erachten die Autorenteams [3, 16] die aktive Beteiligung der Zielgruppe an der Forschung im Rahmen von Patient Public Involvement and Engagement und entsprechen damit sowohl den Forderungen von Alzheimer Europe [12] als auch der nationalen Demenzstrategie [11]. Sollen museumsbasierte Programme von MmD und ihren primären Betreuungspersonen als öffentliche Ressource [23], insbesondere im Rahmen des Social Prescribing [18], genutzt werden, kann die Verknüpfung von Mixed-Methods-Designs mit dem Ansatz des Patient Public Involvement and Engagement [15] einen zusätzlichen Vorteil für die Nutzbarkeit und Zugänglichkeit museumsbasierter Angebote für Menschen mit Demenz und ihre primären Betreuungspersonen bieten [15, 16].

### Limitationen

Umbrella Reviews führen die Ergebnisse bereits bestehender systematischer Reviews und Metaanalysen zusammen. Den dieser Arbeit zugrunde liegenden Übersichtsarbeiten ist gemeinsam, dass sie ihrerseits sehr heterogene Einzelstudien zusammenfassen. Die durchwegs vielversprechenden Ergebnisse qualitativer und teilweise signifikanten Ergebnisse quantitativer Einzelstudien, die in den Übersichtsarbeiten insbesondere zu psychosozialen Aspekten hervorgehoben werden [3, 7, 8, 16, 23], erhalten damit durch die methodischen Besonderheiten eines Umbrella Review möglicherweise nicht das ihnen zustehende Gewicht [9]. Das betrifft insbesondere ihre Relevanz für die aktuelle Versorgungspraxis. Fünf der 6 zugrunde liegenden Übersichtsarbeiten fassen die Ergebnisse zu museumsbasierten Angeboten in gesonderten Abschnitten von übergeordneten kulturbasierten systematischen Reviews zusammen. Von dieser Datenbasis ist kein konsistenter Nachweis der Wirksamkeit museumsbasierter Angebote ableitbar. Neben den bereits oben angeführten Überlegungen zu Qualität und Bias stellt dies eine Limitation des vorliegenden Umbrella Review dar.

## Fazit

- Aktuelle Forschungsergebnisse liefern inkonsistente Hinweise für signifikante Verbesserungen kognitiver und psychosozialer Funktionen bei MmD durch museumsbasierte Angebote.
- Grundlegend festgestellte Verbesserungstendenzen hinsichtlich dieser Funktionen und dementsprechende Ergebnisse qualitativer Studien lassen den Schluss zu, dass gemeindenahe, museumsbasierte (Gruppen)Angebote für MmD und ihre primären Betreuungspersonen eine Ressource für Wohlbefinden, Lebensqualität, Kommunikation und Teilhabe im gemeinsamen Alltagsleben darstellen können.
- Zur Verankerung gemeindenaher, museumsbasierter Angebote als „komplexe Intervention“ in Public-Health-Programmen und Social Prescribing verfolgt die zukünftige Forschung Ansätze wie Patient and Public Involvement and Engagement und entwickelt neben sensiblen, interventionsangepassten Messinstrumenten angemessene Mixed-Methods-Designs.

## Supplementary Information

Online-Supplement 1 Ein- und Ausschlusskriterien

Online-Supplement 2 Data Extraction

Online-Supplement 3 Critical Appraisal
