# Supplementary material for: Effects of museum-based community programs on people with dementia and their primary caregivers—An umbrella review
Source: Z Gerontol Geriatr. 2024 Dec 2;58(1):33–9. [Article in German] doi: 10.1007/s00391-024-02377-2 (PMC11779787; doi:10.1007/s00391-024-02377-2)
Supplement: Supplementary file 1 — Online-Supplement 1 Ein- und Ausschlusskriterien [file 391_2024_2377_MOESM1_ESM.docx]

Ein- und Ausschlusskriterien (Online-Supplement1)

| **Einschlusskriterien** |  |
| --- | --- |
| Zielgruppe/Population/Setting | - In der Gemeinde lebende Menschen mit Demenz/progredienten neurokognitiven Einschränkungen > 50 Jahre - In der Gemeinde lebende Menschen mit Demenz/progredienten neurokognitiven Einschränkungen und ihre primären Betreuungspersonen > 50 Jahre - In Langzeitbetreuungseinrichtungen oder betreuten Wohneinheiten lebende Menschen mit Demenz/progredienten neurokognitiven Einschränkungen unterschiedlicher Ätiologie > 50 Jahre - In Langzeitbetreuungseinrichtungen oder betreuten Wohneinheiten lebende Menschen mit Demenz/progredienten neurokognitiven Einschränkungen unterschiedlicher Ätiologie zusammen mit informellen Betreuungspersonen |
| Diagnosen/  Krankheitsbilder | - Demenzen/progrediente neurokognitive Einschränkungen unterschiedlicher Ätiologie |
| Fokus der Reviews | - Gemeindenahe Angebote in Museen oder in Zusammenarbeit mit Museen und/oder Kunstgalerien und/oder Kunst- und Kulturvermittlungsinstitutionen - Überblicksarbeiten über psychosoziale, nicht-pharmakologische Angebote einschließlich Angebote in Museen oder in Zusammenarbeit mit Museen und/oder Kunstgalerien und/oder Kunst- und Kulturvermittlungsinstitutionen - Museums- und kunstbezogene Angebote aus dem (mittel)europäischen Kulturkreis |
| Outcomes | - Psychosoziale Aspekte - Kognition - Partizipation und Teilhabe - Lebensqualität - Wohlbefinden |
| Publikationsformen | - Systematischer Review - Meta-Analyse - Systematischer qualitativer Review |
| **Ausschlusskriterien** |  |
| Zielgruppe/Population/Setting | - Stationäre Akutbehandlung im Krankenhaus - Museumsmitarbeitende, kunsttherapeutisches & Gesundheitspersonal - Menschen mit Demenz/progredienten neurokognitiven Einschränkungen unterschiedlicher Ätiologie unter 50   Jahren |
| Diagnosen/  Krankheitsbilder | - Neurokognitive Einschränkungen nach Schlaganfall, Schädel-Hirn-Trauma, intrakraniellen Blutungen |
| Fokus der Reviews | - Angebote zur Prävention von Demenz/progredienten neurokognitiven Einschränkungen - Ausschließlich kunsttherapeutische Angebote mit und ohne Zusammenarbeit mit Museen - Ausschließlich Musik- und gartentherapeutische Angebote und Interventionen - Ausschließlich Sport- und bewegungstherapeutische Angebote und Interventionen - Tiergestützte Therapien und Interventionen - Überblicksarbeiten über nicht-pharmakologische Angebote ohne bzw. mit unterrepräsentiertem Bezug zu Museen oder Kunstgalerien - Interventionen aus vorwiegend nicht-europäischen kulturellen Kontexten |
| Outcomes | Ausschließlich   - biomechanische Aspekte und/oder - sensomotorische Aspekte |
| Publikationsformen | - Realist Review - Scoping Review - Thematic Review - Rapid Review - Narrative Review |
