# Supplementary material for: Effects of museum-based community programs on people with dementia and their primary caregivers—An umbrella review
Source: Z Gerontol Geriatr. 2024 Dec 2;58(1):33–9. [Article in German] doi: 10.1007/s00391-024-02377-2 (PMC11779787; doi:10.1007/s00391-024-02377-2)
Supplement: Supplementary file 3 — Online-Supplement 3 Critical Appraisal [file 391_2024_2377_MOESM3_ESM.docx]

Critical Appraisal (Online-Suppelemt 3)

| Letrondo et al, 2023 (Systematic Review) | | | | | | | | | | | | |
| --- | --- | --- | --- | --- | --- | --- | --- | --- | --- | --- | --- | --- |
|  | 1 Review question clearly & explicitly stated? | 2 Inclusion criteria appropriate for review question? | 3 Search strategy appropriate? | 4 Sources and resources used to search for studies adequate? | 5 Criteria for appraising studies appropriate? | 6 ritical appraisal conducted by two or more reviewers independently? | 7 Methods to minimize errors in data extraction? | 8 Methods used to combine studies appropriate? | 9 Likelihood of publication bias assessed? | 10 Recommendations for policy and/or practice supported by the reported data? | 11 Specific directives for new research appropriate? | Include/  exclude? |
| MD | N | Y | Y | Y | Y | Y | Y | Y | U | Y | Y | Y |
| VT | N | Y | Y | Y | Y | Y | Y | Y | U | Y | Y | Y |
| MKP | N | Y | Y | Y | Y | Y | Y | y | U | Y | Y | Y |

| Zeilig et al., 2022 (Systematic Review) | | | | | | | | | | | | |
| --- | --- | --- | --- | --- | --- | --- | --- | --- | --- | --- | --- | --- |
|  | 1 Review question clearly & explicitly stated? | 2 Inclusion criteria appropriate for review question? | 3 Search strategy appropriate? | 4 Sources and resources used to search for studies adequate? | 5 Criteria for appraising studies appropriate? | 6 Critical appraisal conducted by two or more reviewers independently? | 7 Methods to minimize errors in data extraction? | 8 Methods used to combine studies appropriate? | 9 Likelihood of publication bias assessed? | 10 Recommendations for policy and/or practice supported by the reported data? | 11 Specific directives for new research appropriate? | Include/  exclude? |
| MD | N | Y | U | Y | Y | U | U | Y | N | Y | Y | Y |
| VT | N | Y | U | Y | Y | U | U | Y | N | Y | Y | Y |
| MKP | N | Y | U | Y | Y | U | U | Y | N | Y | Y | Y |

| Delfa Lobato at al., 2021 (Systematic Review) | | | | | | | | | | | | |
| --- | --- | --- | --- | --- | --- | --- | --- | --- | --- | --- | --- | --- |
|  | 1 Review question clearly & explicitly stated? | 2 Inclusion criteria appropriate for review question? | 3 Search strategy appropriate? | 4 Sources and resources used to search for studies adequate? | 5 Criteria for appraising studies appropriate? | 6 Critical appraisal conducted by two or more reviewers independently? | 7 Methods to minimize errors in data extraction? | 8 Methods used to combine studies appropriate? | 9 Likelihood of publication bias assessed? | 10 Recommendations for policy and/or practice supported by the reported data? | 11 Specific directives for new research appropriate? | Include/  exclude? |
| MD | N | Y | Y | Y | Y | N | Y | U | N | Y | Y | Y |
| VT | N | Y | Y | Y | Y | N | Y | U | N | Y | Y | Y |
| MKP | N | Y | Y | Y | Y | N | Y | U | N | Y | Y | Y |

| Bourne et al., 2021 (Systematic Review) | | | | | | | | | | | | |
| --- | --- | --- | --- | --- | --- | --- | --- | --- | --- | --- | --- | --- |
|  | 1 Review question clearly & explicitly stated? | 2 Inclusion criteria appropriate for review question? | 3 Search strategy appropriate? | 4 Sources and resources used to search for studies adequate? | 5 Criteria for appraising studies appropriate? | 6 Critical appraisal conducted by two or more reviewers independently? | 7 Methods to minimize errors in data extraction? | 8 Methods used to combine studies appropriate? | 9 Likelihood of publication bias assessed? | 10 Recommendations for policy and/or practice supported by the reported data? | 11 Specific directives for new research appropriate? | Include/  exclude? |
| MD | N |  | Y | Y | Y | Y | U | N | N | Y | Y | Y |
| VT | N | Y | Y | Y | Y | Y | U | N | N | Y | Y | Y |
| MKP | N | Y | Y | Y | Y | Y | U | N | N | Y | Y | Y |

| Young et al., 2016 (Systematic Review) | | | | | | | | | | | | |
| --- | --- | --- | --- | --- | --- | --- | --- | --- | --- | --- | --- | --- |
|  | 1 Review question clearly & explicitly stated? | 2 Inclusion criteria appropriate for review question? | 3 Search strategy appropriate? | 4 Sources and resources used to search for studies adequate? | 5 Criteria for appraising studies appropriate? | 6 Critical appraisal conducted by two or more reviewers independently? | 7 Methods to minimize errors in data extraction? | 8 Methods used to combine studies appropriate? | 9 Likelihood of publication bias assessed? | 10 Recommendations for policy and/or practice supported by the reported data? | 11 Specific directives for new research appropriate? | Include/  exclude? |
| MD | N | Y | Y | Y | Y | U | U | Y | N | Y | Y | Y |
| VT | N | Y | Y | Y | Y | U | U | Y | N | Y | Y | Y |
| MKP | N | Y | Y | Y | Y | U | U | Y | N | Y | Y | Y |

| Delfa-Lobato, et al., 2023 (Meta-Anayse) | | | | | | | | | | | | |
| --- | --- | --- | --- | --- | --- | --- | --- | --- | --- | --- | --- | --- |
|  | 1 Review question clearly & explicitly stated? | 2 Inclusion criteria appropriate for review question? | 3 Search strategy appropriate? | 4 Sources and resources used to search for studies adequate? | 5 Criteria for appraising studies appropriate? | 6 Critical appraisal conducted by two or more reviewers independently? | 7 Methods to minimize errors in data extraction? | 8 Methods used to combine studies appropriate? | 9 Likelihood of publication bias assessed? | 10 Recommendations for policy and/or practice supported by the reported data? | 11 Specific directives for new research appropriate? | Include/  exclude? |
| MD | Y | Y | Y | Y | Y | U | Y | Y | N | U | N | Y |
| VT | Y | Y | Y | Y | Y | U | Y | Y | N | U | N | Y |
| MKP | Y | Y | Y | Y | Y | U | Y | Y | N | U | N | Y |

Critical Appraisal (Supplement 3)
