# Supplementary material for: Effects of museum-based community programs on people with dementia and their primary caregivers—An umbrella review
Source: Z Gerontol Geriatr. 2024 Dec 2;58(1):33–9. [Article in German] doi: 10.1007/s00391-024-02377-2 (PMC11779787; doi:10.1007/s00391-024-02377-2)
Supplement: Supplementary file 3 [file 391_2024_2377_MOESM2_ESM.docx]

Dataextraction (Online-Supplement 2)

Autor:innen (Jahr), Land	Titel	Forschungsfrage/Ziel	Methode	Anzahl einbezogene museums- und kunstbasierte Studien/Gesamtzahl kulturbasierte Studien	Kontext	Population
Letrondo et al. (2023), UK	Systematic review of arts and culture-based interventions for people living with dementia and their caregivers.	Ziele: Lücke in bestehenden Reviews zum Schwerpunkt Kunst und Kultur für MmD schließen; Interventionen überprüfen auf Effekte auf Kognition von MmD sowie Wohlbefinden von MmD & PBP.	Systematischer Review	17/34	Musee, Kunstgalerien, Volkskundemuseen; lokale Kunstschaffende, Ehrenamtliche mit Zusatzausbildungen f. MmD	MmD, PBP hauptsächl lebend; geringe Zahl a
Delfa-Lobato, et al. (2023), Spanien	Benefits of Cultural Activities on People with CognitiveImpairment: A Meta-Analysis.	Ziel: lokalisieren und analysieren von kulturbasierten Studien, um ihren Nutzen für Menschen mit kognitiven Einschränkungen zu identifizieren, zu beschreiben und zu evaluieren.	Meta-Analyse	9/26	Museen oder vergleichbare Kulturinstitutionen mit Zugang zu Kunstgegenständen	Menschen mit neurok Beeinträchtigungen (N mittlerer Schweregrad unterschiedlicher Ätio Alzheimer, nicht näher Demenz, MCI, NCI ass Hochaltrigkeit);
Zeilig, et al. (2022), UK	The psychological and social impacts of museumbased programmes for people with a mild-to-moderate dementia: a systematic review.	Ziel: Erforschen und Identifizieren von psychologischen und sozialen Auswirkungen v. museumsbasierten Programmen f. MmD	Systematischer Review	11/11	öffentl. Museen oder Kunstgalerien	MmD; Menschen m. N vergleichbarer Auswir Demenz; vereinzelt Ei
